# Dor neuropática em pacientes que serão submetidos à artroplastia total primária do joelho: Prevalência e fatores de risco

**DOI:** 10.1055/s-0046-1820457

**Published:** 2026-06-08

**Authors:** Gustavo Waldolato Silva, Rafael Silva E Castro, Gabriela Vieira Brito, Geovanna Reis

**Affiliations:** 1 Departamento de Ortopedia, Hospital Universitário Ciências Médicas, Belo Horizonte, MG, Brasil; 2 Faculdade Ciências Médicas de Minas Gerais, Belo Horizonte, MG, Brasil

**Keywords:** artroplastia do joelho, dor crônica, fatores de risco, neuralgia, prevalência, arthroplasty, replacement knee, chronic pain, neuralgia, prevalence, risk factors

## Abstract

**Objetivo:**

Avaliar a prevalência e os fatores de risco de dor de característica neuropática (DN) em pacientes que serão submetidos a artroplastia total de joelho (ATJ) primária.

**Métodos:**

Trata-se de um estudo prospectivo observacional realizado com pacientes acima de 18 anos, candidatos a ATJ, durante o período de outubro de 2023 a outubro de 2024, em um hospital universitário. A coleta de dados foi realizada baseada no prontuário dos pacientes e na aplicação de um questionário com variáveis socioeconômicas, comorbidades, tratamentos prévios e o Douleur Neuropathique en 4 (DN4i), instrumento validado de triagem para DN. Para avaliar e correlacionar as amostras pareadas foram utilizados os testes de Wilcoxon, Qui-quadrado de independência e exato de Fisher, considerando nível de significância de *p* < 0,05.

**Resultados:**

Dos 68 participantes, 20 (29%) apresentaram resultado positivo para DN. Dentre eles, a maioria era do sexo feminino (80%, *p* = 0,015). A intensidade da dor foi significativamente maior nesse grupo, com 90% relatando EVA entre 8 e 10. Não foram observadas associações significativas com obesidade, diabetes ou uso de moduladores da dor. A qualidade de vida foi mais impactada nos pacientes com DN, que relataram pior estado geral de saúde e maior limitação em atividades físicas e sociais.

**Conclusão:**

A DN é altamente prevalente em pacientes candidatos à ATJ, principalmente do sexo feminino, e está associada a pior qualidade de vida e maior intensidade de dor. A triagem sistemática com o DN4i pode otimizar o diagnóstico e contribuir para estratégias terapêuticas personalizadas.

## Abstract (en)

**Objective:**

This study aimed to evaluate the prevalence and risk factors of neuropathic pain (NP) in patients undergoing primary total knee arthroplasty (TKA).

**Methods:**

This prospective observational study included patients aged 18 years or older who were candidates for TKA, conducted between October 2023 and October 2024 at a university hospital. Data were collected from medical records and through the application of a questionnaire assessing socioeconomic variables, comorbidities, prior treatments, and the interview version of the Douleur Neuropathique in 4 questions (DN4i) questionnaire, a validated screening instrument for NP. Paired samples were analyzed using the Wilcoxon, Chi-squared of independence, and Fisher's exact tests, with a significance level set at *p* < 0.05.

**Results:**

Of the 68 participants, 20 (29%) tested positive for NP. Among these, most were female (80%, *p* = 0.015). Pain intensity was significantly higher in this group, with 90% reporting VAS scores between 8 and 10. There were no significant associations with obesity, diabetes, or the use of pain modulators. Quality of life was more adversely affected in patients with NP, who reported poorer general health status and greater limitations in physical and social activities.

**Conclusion:**

There is a high prevalence of NP among patients undergoing TKA, particularly in females, and is associated with worse quality of life and greater pain intensity. Systematic screening using the DN4i questionnaire may improve diagnosis and support the development of individualized therapeutic strategies.

## Introdução

A dor neuropática (DN) é uma síndrome crônica definida como “dor que surge como consequência direta de uma lesão ou doença que afeta o sistema somatossensorial”. 1 Ela se difere da dor nociceptiva por possuir características como disestesias, sensações elétricas e de queimação. 2 Estima-se que, no Brasil, sua prevalência seja de 14,5% nos pacientes com dor crônica. 3

Dados da literatura apontam o sexo feminino, a idade avançada e o uso de moduladores da dor como fatores associados a DN. 4 5 6 Em pacientes com diabetes mellitus (DM), o controle glicêmico adequado reduz o risco do desenvolvimento da DN e de seus impactos. 7

Entre as ferramentas de rastreio para a DN, está o Douleur Neuropathique en in 4 4 Interview (DN4i), uma versão do DN4, mas que engloba apenas a parte da entrevista, o que possibilita a sua aplicação em atendimentos telefônicos. Uma pontuação maior ou igual a 3 no DN4i indica escore positivo para DN. 8 O DN4 apresenta uma maior praticidade em relação aos demais testes por ter um número menor de itens e alta capacidade de discriminar dor neuropática de nociceptiva. 9

A prevalência global de gonartrose, ou osteoartrite (OA) do joelho é estimada em 3,8%. Esta condição, causa uma limitação funcional importante aos pacientes, muito devido a quadros de dor crônica. A OA é mais comum em mulheres e tende a atingir seu pico em torno dos 50 anos de idade. Fatores como envelhecimento populacional, longevidade, obesidade e demanda funcional dos pacientes, impulsionam o procedimento cirúrgico de artroplastia total de joelho (ATJ) primárias. 10 11

A demanda por ATJ primárias nos Estados Unidos está projetada para crescimento de 673% até 2030, atingindo 3,48 milhões de procedimentos anuais. Globalmente, há uma média de 175 procedimentos por 100.000 habitantes. 12

O objetivo das ATJs é devolver a função e aliviar os sintomas álgicos dos pacientes com gonartrose. No entanto, na prática clínica, observamos pacientes com resposta funcional incompleta e com dor residual, mesmo sem intercorrências cirúrgicas. Sendo assim, o objetivo deste trabalho é investigar a prevalência e fatores de risco de dor crônica com característica neuropática em pacientes com gonartrose candidatos para ATJ primária.

## Materiais e Métodos

Trata-se de um estudo prospectivo observacional, que incluiu pacientes candidatos a ATJ primária durante o período de outubro de 2023 a outubro de 2024 em um hospital universitário. O estudo recebeu a aprovação ética do Comitê de Ética e Pesquisa, sob o número CAAE: 76569923.9.0000.5125.

O presente estudo foi baseado em uma amostra por conveniência; todos os pacientes que passaram por consultas médicas com os ortopedistas neste hospital e receberam indicação para realização de ATJ primária foram recrutados. Os critérios de elegibilidade foram idade acima de 18 anos de idade e serem aptos clínicamente à realização da ATJ primária. O estudo excluiu os pacientes que se recusaram a responder a pesquisa, cujo contato via ligação telefônica não foi bem-sucedido, ou que não estavam realizando ATJ primária (ex.: revisão de prótese). O Termo de Consentimento Livre e Esclarecido foi assinado por todos os pacientes incluídos antes da cirurgia.

Apesar de se tratar de uma amostra de conveniência, foi realizado cálculo amostral previamente para estimativa de prevalência. Considerando-se uma prevalência esperada de DN em pacientes com OA do joelho de 25%, erro absoluto máximo de 10% e nível de confiança de 95%, obteve-se um tamanho amostral mínimo de 65 participantes. O número final incluído (68 pacientes) foi, portanto, superior ao mínimo estimado.

Dois médicos ortopedistas do corpo clínico, que realizam prótese de joelho regularmente participaram da elaboração e aplicação de um questionário, via telefonema, que envolvia dados do prontuário médico, assim como variáveis socioeconômicas (escolaridade e local de habitação, tratamentos prévios, como fisioterapia, infiltração, entre outros), uso de medicações para dor e comorbidades. O DN4i, em sua versão validada e traduzida para o português brasileiro, incluindo apenas a parte em forma de entrevista, foi escolhido por possibilitar a sua aplicação por meio de ligação telefônica, o que permitiu a padronização da coleta e a inclusão de todos os participantes. O controle pós-operatório não se realizou no mesmo local e horário. O teste possui sete itens com dois domínios: o primeiro avalia a característica da dor (presença de queimação, sensação de frio doloroso e choque elétrico); e o segundo avalia os sintomas associados e a presença de disestesias (formigamento, sensação de alfinetada e agulhada, adormecimento e coceira). A pontuação do DN4i varia entre 0 e 7, com cada item do questionário sendo respondido por “sim” ou “não”, equivalentes a 1 e 0, respectivamente. Um escore ≥ 3 no DN4i caracteriza a presença de DN. 8

O questionário também envolve a escala visual analógica (EVA) de dor e o Short Form-8 (SF-8). O primeiro consiste em auxiliar na aferição da intensidade da dor, com escores de 0 a 2 caracterizando dor leve, 3 a 7 dor moderada e 8 a 10 dor intensa. 13 O segundo é uma versão abreviada do Short Form-36 e utiliza oito escalas, com o objetivo de avaliar os aspectos físicos e mentais da qualidade de vida do paciente nas últimas 4 semanas. 14

Um pesquisador independente conduziu as análises. Para caracterizar a amostra, calcularam-se estatísticas descritivas com base em todas as variáveis coletadas. Os dados foram apresentados por meio de medidas de tendência central (média ou mediana) para variáveis quantitativas, e de frequência e percentual para variáveis categóricas.

Para garantir a adequação das análises estatísticas ao tipo de dado e às condições do estudo, o teste de soma de pontos de Wilcoxon foi aplicado para comparar amostras pareadas de dados não paramétricos. O teste Qui-quadrado de independência permitiu avaliar a associação entre variáveis categóricas com amostras maiores, enquanto o teste exato de Fisher foi usado em tabelas de contingência com pequenas frequências, garantindo a validade dos resultados mesmo em grupos menores. Em todos os testes estatísticos, o nível de significância foi previamente estabelecido como *p* < 0,05 e intervalos de confiança (ICs) de 95%. Para a análise dos dados foi utilizado o IBM SPSS Statistics Base (IBM Corp.), versão 22.0.

## Resultados

**Fig. 1:**
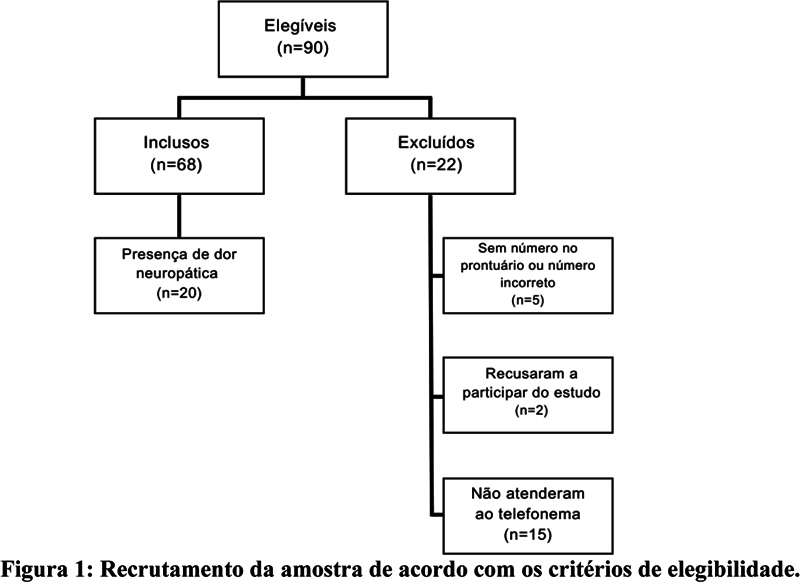
Recrutamento da amostra de acordo com os critérios de elegibilidade.

No total, 90 participantes foram elegíveis para o estudo, dos quais 68 possuíam os dados completos e analisáveis. A disposição da amostra é analisada na Fig. 1 . A mediana de idade foi de 67 anos (intervalo interquartil [IIQ]: 63–73), com 59% tendo ≥ 65 anos, com prevalência do sexo feminino (57%). Obesidade foi identificada em 41% (n = 28) da amostra. As comorbidades mais frequentes foram hipertensão arterial sistêmica (HAS: 76%) e diabetes mellitus (29%). Quanto ao uso de tratamentos farmacológicos, 19% (n = 13) faziam o uso de moduladores da dor, enquanto 72% (n = 49) utilizavam outros tipos de analgésicos. Em relação aos tratamentos prévios, 59% (n = 40) realizaram fisioterapia e 19% (n = 13) relataram infiltração articular. O escore DN4i foi positivo para DN em 29% (n = 20) dos pacientes. As variáveis socioeconômicas e clínicas dos participantes com dados analisáveis estão dispostas na Tabela 1 .

**Tabela 1:** Características sociodemográficas e clínicas da amostra

Características	N = 68 ^a^
**Idade**	67 (63–73)
Maior que 65	40 (59%)
Menor que 65	28 (41%)
**Sexo**	
Feminino	39 (57%)
Masculino	29 (43%)
**Área**	
Belo Horizonte	34 (50%)
Minas Gerais	34 (50%)
**Grau de escolaridade**	
Incompleto	57 (84%)
Superior	11 (16%)
**IMC**	
Não obesos	40 (59%)
Obesos	28 (41%)
**DM**	
Não	48 (71%)
Sim	20 (29%)
**HAS**	
Não	16 (24%)
Sim	52 (76%)
**Uso de moduladores da dor**	
Não	49 (72%)
Sem uso de qualquer medicação	6 (8,8%)
Sim	13 (19%)
**Tratamentos prévios**	
Fisioterapia	40 (59%)
Infiltração	13 (19%)
Ausência de tratamentos prévios	15 (22%)
**Resultado DN4i**	
Negativo	48 (71%)
Positivo	20 (29%)

**Abreviaturas:** DM, diabetes mellitus; DN4i, Douler Neuropathic 4 interview version; HAS, hipertensão arterial sistêmica; IMC, índice de massa corporal.

**Nota:**^*a*^ Valores expressos em mediana (Q1, Q3); n (%).

Entre os pacientes com DN, há maior prevalência no sexo feminino (80%) em comparação aos homens (20%; *p* = 0.015). A maioria dos pacientes relatou intensidade severa de dor de acordo com a EVA (79%), índice ainda maior entre aqueles com DN (90%). No domínio de qualidade de vida, avaliado pelo questionário SF-8, 10% dos pacientes com DN relataram estado de saúde “muito ruim”, enquanto nenhum caso foi registrado no grupo sem DN. Além disso, pacientes com DN apresentaram maior impacto em atividades físicas e sociais. Embora a obesidade tenha sido relatada em 41% dos participantes, não houve associação significativa entre o índice de massa corporal (IMC) e a presença de DN (p = 0.7).

Em relação às comorbidades, não se observou diferença estatisticamente significativa em HAS ( *p* = 0,6) e diabetes mellitus ( *p* ≥ 0,9), entre os grupos com e sem presença de DN. O uso de fármacos moduladores da dor também não demonstrou diferenças estatísticas significativas entre os dois grupos. Todo o cruzamento de dados do escore DN4i com as outras variáveis estão dispostas na Tabela 2 .

**Tabela 2:** Cruzamento de resultado do DN4i com demais variáveis

Características	Total N N = 68 ^a^	Dor neuropática		Valor de *p* ^b^
		Não N = 48 ^a^	Sim N = 20 ^a^	
**Idade**	67 (63–73)	67 (64–73)	67 (63–72)	
Maior que 65	40 (59%)	29 (60%)	11 (55%)	0,8
Menor que 65	28 (41%)	19 (40%)	9 (45%)	0,7
**Sexo**				**0,015**
Feminino	39 (57%)	23 (48%)	16 (80%)	
Masculino	29 (43%)	25 (52%)	4 (20%)	
**Área**				0,3
Belo Horizonte	34 (50%)	22 (46%)	12 (60%)	
Minas Gerais	34 (50%)	26 (54%)	8 (40%)	
**Grau de escolaridade**				> 0,9
Incompleto	57 (84%)	40 (83%)	17 (85%)	
Superior	11 (16%)	8 (17%)	3 (15%)	
**IMC**				0,7
Não obesos	40 (59%)	29 (60%)	11 (55%)	
Obesos	28 (41%)	19 (40%)	9 (45%)	
**Diabetes**				0,6
Não	48 (71%)	33 (69%)	15 (75%)	
Sim	20 (29%)	15 (31%)	5 (25%)	
**HAS**				> 0,9
Não	16 (24%)	11 (23%)	5 (25%)	
Sim	52 (76%)	37 (77%)	15 (75%)	
**Uso de modulador**				> 0,9
Não	49 (72%)	35 (73%)	14 (70%)	
Nenhuma medicação	6 (8,8%)	4 (8,3%)	2 (10%)	
Sim	13 (19%)	9 (19%)	4 (20%)	
**Tratamentos prévios**				> 0.9
Fisioterapia	40 (59%)	28 (58%)	12 (60%)	
Infiltração	13 (19%)	9 (19%)	4 (20%)	
Não	15 (22%)	11 (23%)	4 (20%)	
**EVA**				0,2
Intensa (8–10)	54 (79%)	36 (75%)	18 (90%)	
Moderada (3–7)	14 (21%)	12 (25%)	2 (10%)	
**Q1SF8: Estado de saúde**				0,12
Boa	39 (57%)	29 (60%)	10 (50%)	
Muito ruim	2 (2,9%)	0 (0%)	2 (10%)	
Regular	23 (34%)	17 (35%)	6 (30%)	
Ruim	4 (5,9%)	2 (4,2%)	2 (10%)	
**Q2SF8: Atividade física**				0,7
Impossibilidade de fazer atividades físicas	3 (4.4%)	2 (4.2%)	1 (5.0%)	
Muito pequena	1 (1,5%)	1 (2,1%)	0 (0%)	
Regular	8 (12%)	7 (15%)	1 (5,0%)	
Significativa	56 (82%)	38 (79%)	18 (90%)	
**Q3SF8: Trabalho diário**				0,11
Impossibilidade de trabalho diário	5 (7,4%)	1 (2,1%)	4 (20%)	
Muito pequena	1 (1,5%)	1 (2,1%)	0 (0%)	
Nenhuma	1 (1,5%)	1 (2,1%)	0 (0%)	
Regular	11 (16%)	8 (17%)	3 (15%)	
Significativa	50 (74%)	37 (77%)	13 (65%)	
**Q4SF8: Dor corporal nas últimas 4 semanas**				**0,046**
Branda	17 (25%)	14 (29%)	3 (15%)	
Grave	15 (22%)	8 (17%)	7 (35%)	
Moderada	32 (47%)	25 (52%)	7 (35%)	
Muito grave	4 (5,9%)	1 (2,1%)	3 (15%)	
**Q5SF8: Vitalidade no último mês**				0,2
Alta	30 (44%)	24 (50%)	6 (30%)	
Baixa	11 (16%)	8 (17%)	3 (15%)	
Bem alta	1 (1,5%)	0 (0%)	1 (5.0%)	
Nenhuma	3 (4,4%)	1 (2,1%)	2 (10%)	
Regular	23 (34%)	15 (31%)	8 (40%)	
**Q6SF8: Atividade social**				0,3
Impossibilidade de fazer atividades sociais	2 (2,9%)	1 (2,1%)	1 (5,0%)	
Muito pequena	3 (4,4%)	1 (2,1%)	2 (10%)	
Nenhuma	49 (72%)	35 (73%)	14 (70%)	
Regular	5 (7,4%)	5 (10%)	0 (0%)	
Significativa	9 (13%)	6 (13%)	3 (15%)	
**Q7-SF8: Problema emocional**				> 0,9
Brandos	12 (18%)	9 (19%)	3 (15%)	
Moderados	6 (8,8%)	4 (8,3%)	2 (10%)	
Nenhum	45 (66%)	31 (65%)	14 (70%)	
Significativos	5 (7,4%)	4 (8,3%)	1 (5,0%)	
**Q8:SF8: Limitação das atividades diárias por problemas pessoais ou emocionais**				> 0,9
Muito pequena	4 (5,9%)	3 (6,3%)	1 (5,0%)	
Nenhuma	59 (87%)	42 (88%)	17 (85%)	
Regular	3 (4,4%)	2 (4,2%)	1 (5,0%)	
Significativa	2 (2,9%)	1 (2,1%)	1 (5,0%)	

**Abreviaturas:** DN4i, Douler Neuropathic 4 interview version; EVA, escala visual analógica; HAS, hipertensão arterial sistêmica; IMC, índice de massa corporal; SF-8, Short Form-8.

**Notas:**^*a*^ Mediana (Q1, Q3); n (%). ^*b*^ Teste de soma de postos de Wilcoxon; teste qui-quadrado de independência; teste exato de Fisher.

## Discussão

Este estudo demonstrou que a prevalência de DN em pacientes candidatos à ATJ primária foi de 29%, com predominância significativa no sexo feminino (80%, *p* = 0,015). Além disso, os pacientes com DN relataram maior intensidade de dor, com 90% (n = 18) classificando-a como intensa (EVA: 8–10), e apresentaram maior impacto em qualidade de vida, com 10% (n = 2) relatando estado de saúde “muito ruim” e maiores limitações físicas e sociais.

A alta prevalência de DN neste grupo pode ser explicada pela presença de fatores de risco conhecidos, como o sexo feminino e idade avançada, o que está de acordo com outros estudos epidemiológicos sobre DN. A associação com o sexo feminino, também foi reportada por outras investigações, que atribuem tal prevalência a diferenças hormonais, como o papel dos estrogênios na modulação da dor e sensibilização central aumentada, conforme descrito por Torrance et al. 4 e Bouhassira et al. 5

Entretanto, não foram observadas associações significativas entre a DN e comorbidades como obesidade, HAS ou DM, divergindo de pesquisas que apontam o papel dessas condições como fatores predisponentes. 6 7 15

A intensidade elevada da DN e seu impacto negativo na qualidade de vida corroboram achados da literatura que relacionam essa condição a um maior sofrimento psicológico e funcional. 7 16 17 Essa relação foi observada em estudos que utilizaram questionários como o EuroQol 5-Dimension (EQ-5D) e 12-Item Short Form Health Survey, version 2 (SF-12v2), que possuem o mesmo propósito do SF-8 e apontam piores escores físicos e mentais nos pacientes afetados. 17 O impacto negativo também inclui limitações em atividades diárias e sociais, fatores comumente observados em populações ortopédicas com dor crônica.

Maiores restrições em atividades sociais e diárias reforçam a necessidade de abordagens multidisciplinares para manejo dessa população. Estudos como os de Helito et al. 17 e Zolio et al. 18 enfatizam que a detecção precoce pode orientar estratégias de reabilitação mais eficazes, incluindo fisioterapia especializada e o uso de moduladores neurológicos. Estudos recentes indicam que a prevalência da DN em pacientes com OA varia entre 20 e 28,6%, com impacto significativo na intensidade da dor e na qualidade de vida. 17 Em um estudo multicêntrico, pacientes com DN apresentaram maior comprometimento físico e mental, corroborando os achados de que essa condição é associada a maior limitação funcional e pior desfecho cirúrgico. 18

A elevada proporção de pacientes com dor intensa (EVA: 8–10) no grupo neuropático reforça a maior severidade da DN, já descrita em estudos prévios como fator que amplifica a incapacidade funcional e reduz a qualidade de vida. 4 No contexto da ATJ, esses achados destacam a importância de identificar precocemente pacientes com DN para prevenir impactos negativos nos resultados pós-operatórios. 19

Embora as intervenções não farmacológicas, como fisioterapia, tenham sido amplamente utilizadas, não houve diferença significativa entre os grupos com e sem DN, sugerindo que abordagens adicionais são necessárias para manejar esse subtipo de dor. Estudos sugerem que moduladores de dor, como anticonvulsivantes e antidepressivos, podem ser eficazes no manejo da DN. 4 5 6

Este estudo apresenta algumas limitações. Em primeiro lugar, a coleta de dados foi realizada por meio de entrevistas telefônicas, utilizando o DN4i. Embora o questionário facilite o acesso aos pacientes e padronize a aplicação do instrumento, ele impede a avaliação presencial de sinais neurológicos e pode aumentar o risco de viés de lembrança e de compreensão das questões por parte dos participantes. Além disso, não foram utilizados instrumentos específicos de rastreio cognitivo durante as ligações, o que pode ter levado a respostas menos precisas em pacientes idosos com possível comprometimento cognitivo.

Em segundo lugar, algumas comorbidades potencialmente relacionadas à DN (neuropatia periférica de origem diabética, radiculopatias lombares ou outras doenças do sistema nervoso periférico) podem ter influenciado os escores do DN4i e não foram avaliadas de forma detalhada, o que limita a capacidade de distinguir entre DN atribuível exclusivamente à OA do joelho e outras causas concomitantes. Por fim, o delineamento observacional e a amostra proveniente de um único centro limitam a generalização dos achados, impedindo o estabelecimento de relações causais entre potenciais fatores de risco e a presença de DN.

Os principais pontos fortes observados são do ineditismo na investigação de DN em pacientes pré-operatórios de ATJ primária, pois trata-se de uma característica subdiagnosticada e, consequentemente, subtratada na rotina ortopédica, nessa coorte em especial. Além disso, a utilização de ferramentas diagnósticas que podem ser aplicadas remotamente aos pacientes facilitou o contato e a assiduidade dos pacientes. O DN4i também permite a condução de estudos que comparam desfechos clínicos em diversas regiões.

Estudos futuros, em especial investigações longitudinais, são necessários para melhor avaliar a prevalência e principais fatores que acarretam desfechos funcionais piores em pacientes com DN. Além disso, ampliar o conhecimento epidemiológico acerca dessa condição pode melhorar o prognóstico desses pacientes, com ajuste nas devidas condutas terapêuticas.

## Conclusão

A dor de característica neuropática foi identificada em 29% dos pacientes com gonartrose candidatos ATJ primária, sendo mais frequente no sexo feminino. A presença de DN associou-se a maior intensidade de dor e a piores escores de qualidade de vida, evidenciando que essa condição é um componente relevante do quadro doloroso pré-operatório.

## References

[1] Jensen T S Baron R Haanpää M A new definition of neuropathic pain Pain 2011 152 10 2204 2205 10.1016/j.pain.2011.06.017 21764514

[2] Treede R D Jensen T S Campbell J N Neuropathic pain: redefinition and a grading system for clinical and research purposes Neurology 2008 70 18 1630 1635 10.1212/01.wnl.0000282763.29778.59 18003941

[3] Udall M Kudel I Cappelleri J C Epidemiology of physician-diagnosed neuropathic pain in Brazil J Pain Res 2019 12 243 253 10.2147/JPR.S160504 30662280 PMC6327897

[4] Torrance N Smith B H Bennett M I Lee A J The epidemiology of chronic pain of predominantly neuropathic origin. Results from a general population survey J Pain 2006 7 04 281 289 10.1016/j.jpain.2005.11.008 16618472

[5] Bouhassira D Lantéri-Minet M Attal N Laurent B Touboul C Prevalence of chronic pain with neuropathic characteristics in the general population Pain 2008 136 03 380 387 10.1016/j.pain.2007.08.013 17888574

[6] Waldolato G Polese J C Pires R E The hidden impact of neuropathic pain after surgical fixation of wrist, hip, and ankle fractures: A cross-sectional retrospective study evaluating its prevalence and risk factors Injury 2023 54 06 110708 10.1016/j.injury.2023.03.036 38143148

[7] Smith B H Torrance N Bennett M I Lee A J Health and quality of life associated with chronic pain of predominantly neuropathic origin in the community Clin J Pain 2007 23 02 143 149 10.1097/01.ajp.0000210956.31997.89 17237663

[8] Bouhassira D Attal N Alchaar H Comparison of pain syndromes associated with nervous or somatic lesions and development of a new neuropathic pain diagnostic questionnaire (DN4) Pain 2005 114(1-2):29 36 10.1016/j.pain.2004.12.010 15733628

[9] Santos J G Brito J O Andrade D C Translation to Portuguese and validation of the Douleur Neuropathique 4 questionnaire J Pain 2010 11 05 484 490 10.1016/j.jpain.2009.09.014 20015708

[10] American Physical Therapy Association Jette D U Hunter S J Burkett L Physical Therapist Management of Total Knee Arthroplasty Phys Ther 2020 100 09 1603 1631 10.1093/ptj/pzaa099 32542403 PMC7462050

[11] Expert Panel on Musculoskeletal Imaging Walker E A Fox M G Blankenbaker D G ACR Appropriateness Criteria® Imaging After Total Knee Arthroplasty: 2023 Update J Am Coll Radiol 2023 20(11S):S433 S454 10.1016/j.jacr.2023.08.014 38040463

[12] Klug A Gramlich Y Rudert M The projected volume of primary and revision total knee arthroplasty will place an immense burden on future health care systems over the next 30 years Knee Surg Sports Traumatol Arthrosc 2021 29 10 3287 3298 10.1007/s00167-020-06154-7 32671435 PMC7362328

[13] Jensen M P Karoly P Braver S The measurement of clinical pain intensity: a comparison of six methods Pain 1986 27 01 117 126 10.1016/0304-3959(86)90228-9 3785962

[14] ARISCAT Group Vallès J Guilera M Briones Z Gomar C Canet J Alonso J Validity of the Spanish 8-item short-form generic health-related quality-of-life questionnaire in surgical patients: a population-based study Anesthesiology 2010 112 05 1164 1174 10.1097/ALN.0b013e3181d3e017 20418697

[15] Rbia N van der Vlies C H Cleffken B I Selles R W Hovius S ER Nijhuis T HJ High Prevalence of Chronic Pain With Neuropathic Characteristics After Open Reduction and Internal Fixation of Ankle Fractures Foot Ankle Int 2017 38 09 987 996 10.1177/1071100717712432 28670914

[16] Keene D J Knight R Bruce J Chronic pain with neuropathic characteristics after surgery for major trauma to the lower limb: prevalence, predictors, and association with pain severity, disability, and quality of life in the UK WHiST trial Bone Joint J 2021 103-B 06 1047 1054 10.1302/0301-620X.103B.BJJ-2020-2204.R1 33902306

[17] Helito C P Moreira F S Santiago M AM Prevalence and interference of neuropathic pain in the quality of life in patients with knee osteoarthritis Clinics (Sao Paulo) 2023 78 100287 10.1016/j.clinsp.2023.100287 37778166 PMC10757282

[18] Zolio L Lim K Y McKenzie J E Systematic review and meta-analysis of the prevalence of neuropathic-like pain and/or pain sensitization in people with knee and hip osteoarthritis Osteoarthritis Cartilage 2021 29 08 1096 1116 10.1016/j.joca.2021.03.021 33971205

[19] Bertram W Howells N White S P Prevalence and patterns of neuropathic pain in people with chronic post-surgical pain after total knee arthroplasty Bone Joint J 2024 106-B 06 582 588 10.1302/0301-620X.106B6.BJJ-2023-0889.R1 38821515

